# DTI changes of brachial plexus nerve roots in amyotrophic lateral sclerosis and their correlation with electrophysiology

**DOI:** 10.1186/s41747-025-00645-3

**Published:** 2025-11-04

**Authors:** Shanshan Wang, Yuxin Li, Xuewen Xing, Xiao Man, Yufan Chen, Guangbin Wang

**Affiliations:** 1 Department of Radiology, Shandong Provincial Hospital Affiliated to Shandong First Medical University, Jinan, Shandong China; 2 Shandong First Medical University, Jinan, Shandong China; 3 Department of Neurology, Shandong Provincial Hospital Affiliated to Shandong First Medical University, Jinan, Shandong China

**Keywords:** Amyotrophic lateral sclerosis, Brachial plexus, Diffusion tensor imaging, Electrophysiology, Magnetic resonance imaging.

## Abstract

**Background:**

Amyotrophic lateral sclerosis (ALS) is a progressive motor neuron disease with peripheral nerve involvement, but current diagnostics are limited. Diffusion tensor imaging (DTI) may improve microstructural assessment and correlate with clinical markers. We investigated the diffusion properties of the brachial plexus in ALS and examined their relationships with electrophysiological parameters of upper limb nerves.

**Materials and methods:**

We enrolled 25 ALS patients and 22 age- and sex-matched healthy controls. DTI of the brachial plexus was conducted to measure fractional anisotropy (FA), mean diffusivity (MD), axial diffusivity (AD), and radial diffusivity (RD). Differences in DTI parameters between the two groups were analyzed. Correlations between DTI parameters and ALS Functional Rating Scale-Revised (ALSFRS-R) scores, along with electrophysiological measurements, were assessed.

**Results:**

In ALS patients compared to controls, FA and AD values were significantly lower (*p* ≤ 0.002), while the RD value was significantly higher (*p* = 0.002). There were no statistically significant differences in MD (*p* = 0.540). Both FA and AD showed a positive correlation with ALSFRS-R score, ALSFRS-upper limb score, and compound muscle action potential amplitude of median, ulnar, and radial nerves (*r* ≥ 0.480; *p* ≤ 0.015). The RD values showed a negative correlation with ALSFRS-upper limb score and motor nerve conduction velocity of median, ulnar, and radial nerves (*r* ≤ -0.415; *p* ≤ 0.039).

**Conclusion:**

FA, AD, and RD values of DTI showed the potential to identify microstructural changes in the brachial plexus nerve roots of ALS patients and may serve as potential indicators of nerve conduction function in the upper extremities.

**Relevance statement:**

DTI may reveal microstructural changes in ALS brachial plexus, correlating with nerve dysfunction, offering novel biomarkers for evaluation of upper limb neurodegeneration.

**Key Points:**

- Lower Fractional anisotropy (FA) and axial diffusivity (AD), and higher radial diffusivity (RD) were shown in amyotrophic lateral sclerosis (ALS) brachial plexus.
- Diffusion tensor imaging (DTI) parameters correlated with clinical and electrophysiological parameters.
- FA, AD, and RD detected ALS nerve microstructural changes, indicating abnormal conduction function.

**Graphical Abstract:**

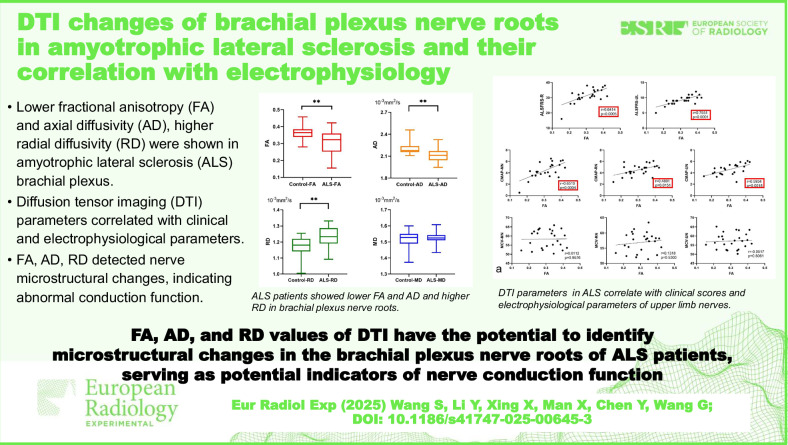

## Background

Amyotrophic lateral sclerosis (ALS) is a progressive and chronic neurodegenerative disorder that impacts both upper and lower motor neurons [1]. Clinical manifestations include progressive muscle atrophy, weakness, and pyramidal signs, ultimately leading to difficulty in swallowing and respiratory muscle weakness, resulting in death [1]. Recent studies suggest that the peripheral nervous system plays a significant role in the pathogenic progression of ALS, particularly during the early stages. This includes processes such as axonal degeneration and Wallerian degeneration of nerve fibers [2–4].

The diagnosis of ALS primarily relies on medical history and clinical examination; however, these methods are limited in sensitivity and specificity, particularly during the early stages of the disease [1]. Neurologic and electrophysiological examination provides information about damage to the peripheral nervous system, which is the most commonly used method for diagnosing ALS [5]. However, such examinations are time-consuming, subject to operator proficiency and subjectivity, and are invasive and painful to patients, with low specificity and sensitivity for ALS diagnosis [6, 7]. There is a need for a new methodology to assess microstructural changes of peripheral nerves in ALS and to eventually improve assessment.

In recent years, magnetic resonance neurography has provided a non-invasive and effective means for displaying peripheral nerve anatomy and pathological changes. Prior studies showed nerve bundle swelling and increased T2WI signals in the bilateral brachial plexus nerves of ALS patients, further confirming the involvement of the peripheral nervous system in ALS [8, 9].

Diffusion tensor imaging (DTI) is an advanced magnetic resonance imaging technique that offers quantitative information regarding changes in neuronal microstructure. DTI is primarily used to capture the anisotropic diffusion properties of water molecules within tissues, through the measurement of fractional anisotropy (FA), mean diffusivity (MD), axial diffusivity (AD), and radial diffusivity (RD) [10]. DTI is increasingly widely used in the peripheral nervous system [11]. The reliability and repeatability of DTI sequences in the peripheral nerve were confirmed in prior studies [12]. Studies by Michael et al [13] in a group of patients with multiple peripheral nerve lesions and healthy volunteers also confirmed that DTI can effectively improve the diagnostic rate for peripheral nerve lesions. Currently, there is limited research on the use of DTI to investigate peripheral nerves in ALS patients, and the relationship between DTI parameters and electrophysiological markers remains poorly explored.

This study, therefore, aimed to examine the microstructural characteristics of the brachial plexus nerve roots in ALS patients by quantifying DTI parameters, while also investigating the correlations between these DTI measures and clinical as well as electrophysiological markers of nerve function.

## Materials and methods

### Subjects

This prospective study was approved by the institutional ethics committee, and all participants provided written informed consent. A total of 25 patients with upper extremity onset of ALS and 22 age- and gender-matched healthy controls were enrolled consecutively between December 2018 and July 2023. Each participant underwent a neurological and electrophysiological assessment conducted by a neurologist specializing in peripheral nerve disorders, who has 23 years of experience in the field of neurology. All ALS patients had the ALS Functional Rating Scale-Revised (ALSFRS-R) assessment on the same day that magnetic resonance imaging (MRI) was performed. Inclusion criteria were a diagnosis of ALS according to the Gold Coast criteria [1]. The healthy control group had no history of neurological, psychiatric, or significant medical conditions.

### Clinical and electrophysiological data acquisition

For ALS patients, functional status was assessed using ALSFRS-R, scored from 0 to 48 by a neurologist specialized in ALS with 10 years of experience. The upper limb score, known as ALSFRS-UL, was determined by summing items 4, 5, and 6 of the ALSFRS-R, with a total possible score of 12 points.

Prior to the MRI scan, all ALS patients underwent neurological and electrophysiological evaluations conducted by a skilled electromyographer with 12 years of experience, with the skin temperature of the upper limbs maintained at 36 °C. The compound muscle action potential (CMAP) amplitude and motor nerve conduction velocity (MCV) were measured for the median nerve (MN), ulnar nerve (UN), and radial nerve (RN), and these values were subsequently used for correlation analyses. The reference values established by Preston and Shapiro [14] were applied in the analysis.

### MRI protocol

MRI scans for both ALS patients and healthy controls were conducted using a 3.0-T scanner (Ingenia, Philips Healthcare) equipped with a 16-channel neurovascular coil. The imaging sequences included DTI and three-dimensional SHeath signal increased with INKed rest-tissue RARE Imaging (SHINKEI).

The DTI sequence was acquired in the axial plane, covering the entire bilateral C5–T1 nerve roots. The scanning parameters were as follows: repetition time = 4,892 ms, echo time = 103 ms; diffusion-sensitive gradient directions = 32, *b*-value = 800 s/mm², flip angle = 90°, slice thickness = 3 mm, interslice gap = 0 mm; field of view = 300 × 180 mm^2^; sensitive encoding acceleration factor = 2.3; acquisition matrix = 128 × 128, number of excitation = 2; acquisition time = 6:34 min:s.

Three-dimensional SHINKEI was performed with the following parameters: repetition time = 2,200 ms, echo time 250 ms; field of view = 280 × 400 mm^2^; echo train length 100, acquisition matrix  = 232 × 321; voxel size 0.45 × 0.45 × 1.0 mm³, inversion-prepared motion-sensitized driven equilibrium−iMSDE duration = 35 ms, phase encoding direction from right to left; phase oversampling = 50%; acquisition time = 6:6 min:s.

### Image post-processing

DTI post-processing was performed using the Philips ISP workstation. DTI images were reviewed by two radiologists with 5 and 8 years of experience in neuroradiology, who were blinded to the subjects’ details. To ensure accurate localization of the brachial plexus nerves, high-resolution three-dimensional SHINKEI images—which provide excellent structural delineation of neural anatomy—were fused with the DTI datasets. This fusion allowed for precise anatomical correlation between the detailed nerve visualization of three-dimensional SHINKEI and the diffusion metrics derived from DTI. For each participant, regions of interest (ROIs) were systematically placed along the bilateral C5–C8 nerve roots. At each nerve root, three separate ROIs were defined on axial DTI parameter maps (FA, MD, AD, and RD) at standardized distances: 1 cm, 2 cm, and 3 cm distal to the dorsal root ganglion. This multilevel approach ensured comprehensive coverage of the nerve segments while accounting for potential regional variations in diffusion properties. At each predefined level (1 cm, 2 cm, and 3 cm), the radiologists maximized ROI size to fully encompass the cross-sectional area of the nerve while rigorously excluding adjacent confounding structures such as blood vessels, fat, and surrounding connective tissues.

**Fig. 1:**
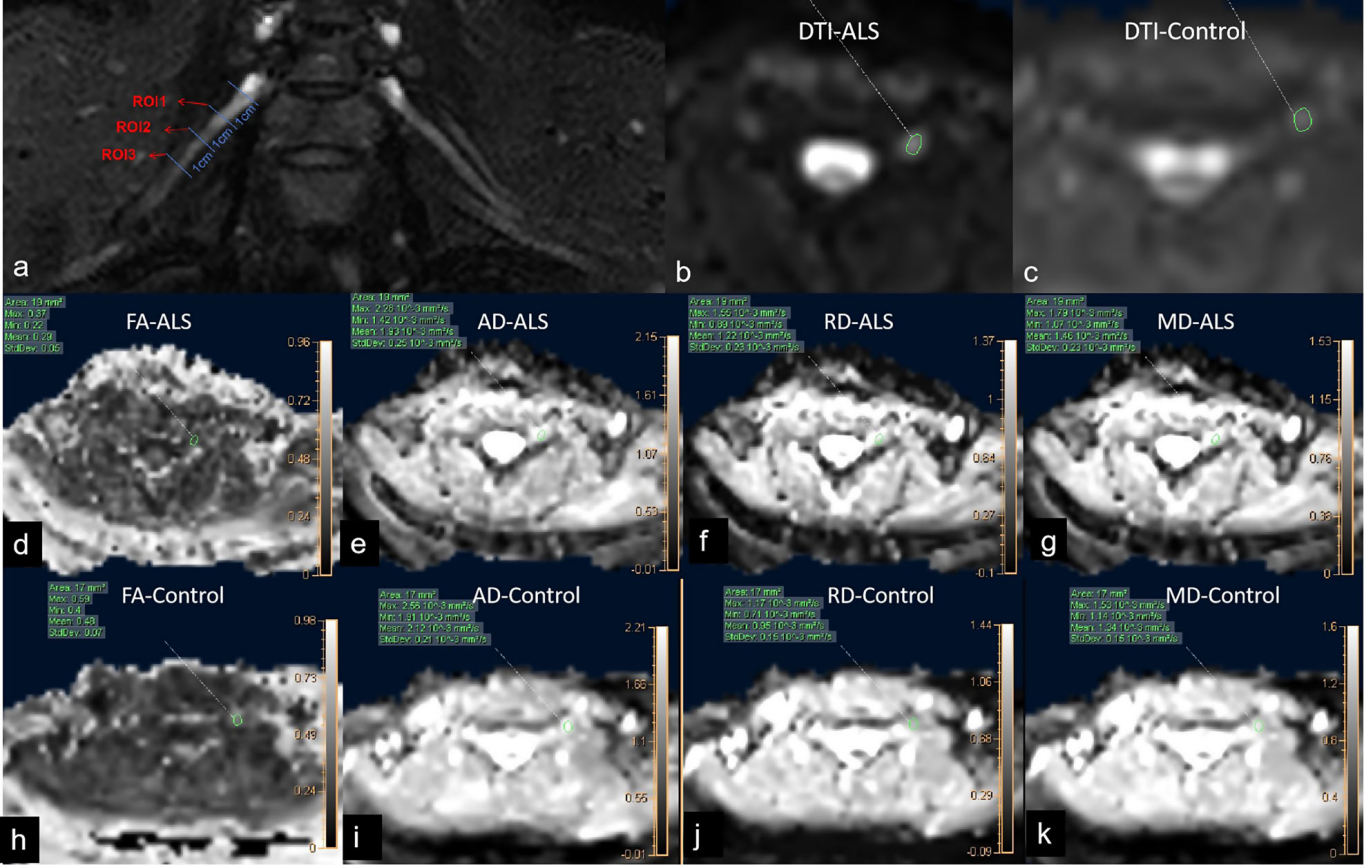
**a** ROI placement: three ROIs were placed at 1, 2, and 3 cm from the ganglion on each nerve root to measure the DTI parameter values of the nerve. **b** DTI raw image of an ALS patient. **c** DTI raw image of a healthy control. (**d**–**g**) FA, AD, RD, and MD parameter maps of the ALS patient. (**h**–**k** FA, AD, RD, and MD parameter maps of the healthy control. AD, Axial diffusivity; ALS, Amyotrophic lateral sclerosis; DTI, Diffusion tensor imaging; FA, Fractional anisotropy; MD, Mean diffusivity; RD, Radial diffusivity; ROI, Region of interest

Following ROI delineation, the imaging software automatically calculated diffusion metrics (FA, MD, AD, and RD) for each nerve segment. To ensure measurement reliability, the process was repeated three times per ROI, and the mean value was recorded independently by both radiologists. The final reported values represented the average of the measurements obtained by the two observers. In total, this study analyzed 200 nerve roots from 25 ALS patients and 176 nerve roots from age-matched healthy controls, providing a robust dataset for comparative analysis. The measurement method is illustrated in Fig. 1.

### Statistical analyses

Statistical analyses were conducted with Prism Version 8 software, and a *p*-value lower than 0.05 was deemed statistically significant. *χ*^2^ tests were applied to assess gender differences between ALS patients and healthy controls. The distribution of data for age, height, and weight was assessed for normality using the Shapiro-Wilk test and for homogeneity of variances using Levene’s test. Based on these assessments, comparisons were made using independent samples t-tests (for normally distributed data) or Mann-Whitney U tests (for non-normally distributed data).

The consistency of FA, MD, AD, and RD measurements between two observers was assessed using intraclass correlation coefficient analysis, with reliability values referenced according to Landis and Koch [14]. The distribution of data for FA, MD, AD, and RD was assessed for normality using the Shapiro-Wilk test and for homogeneity of variances using Levene’s test. Based on these assessments, comparisons of FA, MD, AD, and RD between ALS patients and healthy controls were made using independent samples t-tests (for normally distributed data) or Mann-Whitney U tests (for non-normally distributed data). Pearson correlation analysis was performed to examine the relationship between the average FA, MD, RD and AD values of C5–C8 nerve roots and ALSFRS-R, ALSFRS-UL scores. Additionally, Pearson correlation was used to assess the correlation between the average FA, MD, AD, and RD values of C5–C8 nerve roots and electrophysiological parameters (CMAP amplitude and MCV) of MN, UN, and RN in ALS patients. Finally, receiver operating characteristic (ROC) analysis was performed to evaluate the diagnostic capability of the FA, MD, AD, and RD values of individual C5–C8 nerve roots, with the area under the ROC curve (AUROC), critical values, sensitivity, and specificity calculated.

## Results

### Clinical data

The ALS group consisted of 15 males and 10 females, aged 61.7 ± 7.2 years (mean ± standard deviation). The disease duration varied widely, ranging from 7 to 68.3 months, with a median of 13 months. No significant differences were observed between ALS patients and healthy controls in terms of age, gender, weight, or height. Clinical data for both the ALS patients and healthy controls are presented in Table 1. The ALSFRS-R score for ALS patients was 32.4 ± 5.0 (mean ± standard deviation), and the ALSFRS-UL score was 9.5 ± 1.5 (mean ± standard deviation).

**Table 1:** Demographic of patients with ALS and healthy controls

Demographic or clinical feature	ALS patients	Healthy controls	*p*-value
Subjects	25	22	
Sex			
Female	10	10	
Male	15	12	
Age at MRI (years)	61.7 ± 7.2	62.5 ± 8.0	0.740
Body weight (kg)	72.4 ± 12.1	68.4 ± 11.8	0.546
Body height (cm)	168.1 ± 7.8	166.2 ± 8.2	0.488
Duration of symptom onset before MRI (range, months)	7–68	--	--
ALSFRS-Revised score	32.4 ± 5.04	--	--
ALSFRS-upper limb score	9.5 ± 1.5	--	--

Data are given as frequencies or mean ± standard deviation

*ALS* Amyotrophic lateral sclerosis, *ALSFRS* ALS Functional Rating Scale, *MRI* Magnetic resonance imaging

### Interobserver agreement of DTI parameters

The intra-observer agreement for FA, MD, AD, and RD, as assessed by the intraclass correlation coefficients, was 0.885 (95% confidence interval: 0.815–0.924), 0.841 (0.720–0.912), 0.852 (0.811–0.908), and 0.873 (0.822–0.973), respectively.

### DTI changes of brachial plexus nerve roots and ROC analysis

**Fig. 2:**
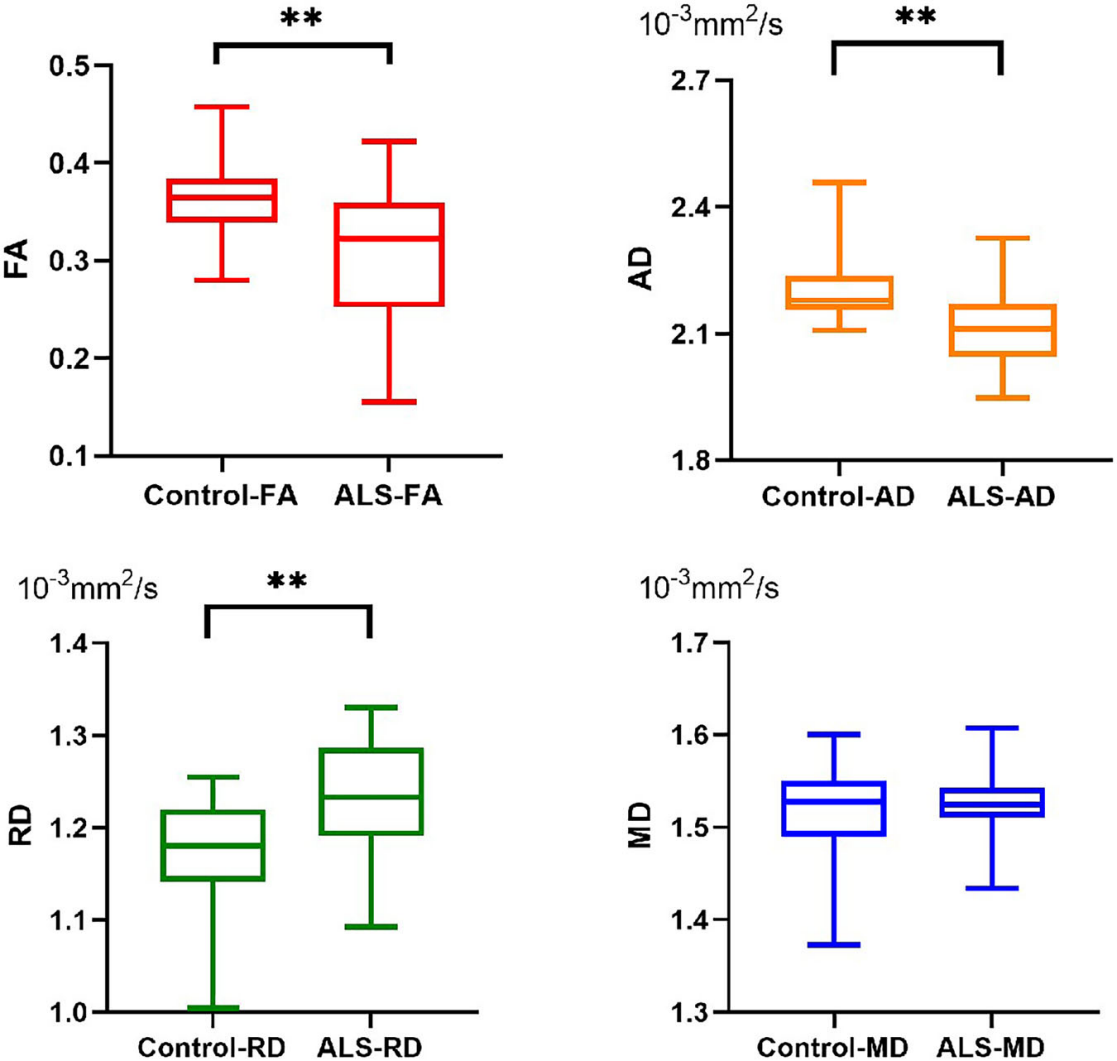
Diffusion tensor imaging parameters of ALS patients and healthy volunteers. *p < 0.05; **p < 0.01. AD, Axial diffusivity; ALS, Amyotrophic lateral sclerosis; FA, Fractional anisotropy; MD, Mean diffusivity; RD, Radial diffusivity

There were no significant differences in FA, MD, AD, or RD values between the left and right C5–C8 nerve roots in both healthy controls and ALS patients (*p* ≥ 0.685). The FA and AD value of C5 − C8 nerve roots were significantly lower in ALS patients than in healthy controls (*p* = 0.001 for FA values, *p* = 0.002 for AD values). The RD value of C5−C8 nerve roots was significantly higher in ALS patients than in healthy controls (*p* = 0.002). There were no statistically significant differences in the MD value of C5–C8 nerve roots between ALS patients and healthy controls (*p* = 0.540). The statistical results are shown in Table 2 and Fig. 2.

**Table 2:** Diffusion tensor imaging parameters of ALS patients and healthy controls

Parameter	ALS patients	Healthy controls	*p*-value
Fractional anisotropy	0.31 ± 0.07	0.37 ± 0.04	0.001
Mean diffusivity (×10^-3^ mm^2^/s)	1.53 ± 0.04	1.52 ± 0.05	0.540
Axial diffusivity (×10^-3^ mm^2^/s)	2.11 ± 0.10	2.20 ± 0.08	0.002
Radial diffusivity (×10^-3^mm^2^/s)	1.23 ± 0.06	1.18 ± 0.06	0.002

*ALS* Amyotrophic lateral sclerosis

**Fig. 3:**
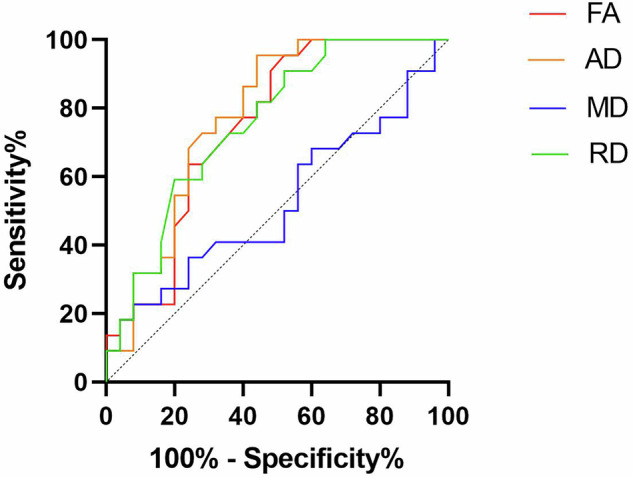
Receiver operating characteristic curves of FA (fractional anisotropy), AD (axial diffusivity), MD (mean diffusivity), and RD (radial diffusivity)

ROC curves showed the optimal diagnostic thresholds, diagnostic sensitivity, specificity, and AUROC for FA, AD, and RD values of C5−C8 brachial plexus nerves in ALS patients (Table 3 and Fig. 3). The diagnostic specificity and sensitivity for FA, AD, and RD were significantly higher compared to those of MD (*p* ≤ 0.004).

**Table 3:** Diagnostic performance of diffusion tensor imaging parameters

Parameter	Sensitivity	Specificity	AUROC	Cutoff value	*p*-value
Fractional anisotropy	68.18%	68.00%	0.7473	0.3463	0.004
Axial diffusivuty	86.36%	60.00%	0.7773	2.130 × 10^-3^ mm^2^/s	0.001
Mean diffusivity	40.91%	72.00%	0.5236	1.514 × 10^-3^ mm^2^/s	0.782
Radial diffusivity	63.64%	72.00%	0.7536	1.199 × 10^-3^ mm^2^/s	0.003

*AUROC* Area under the receiver operating characteristic curve

### Correlation analyses

**Fig. 4:**
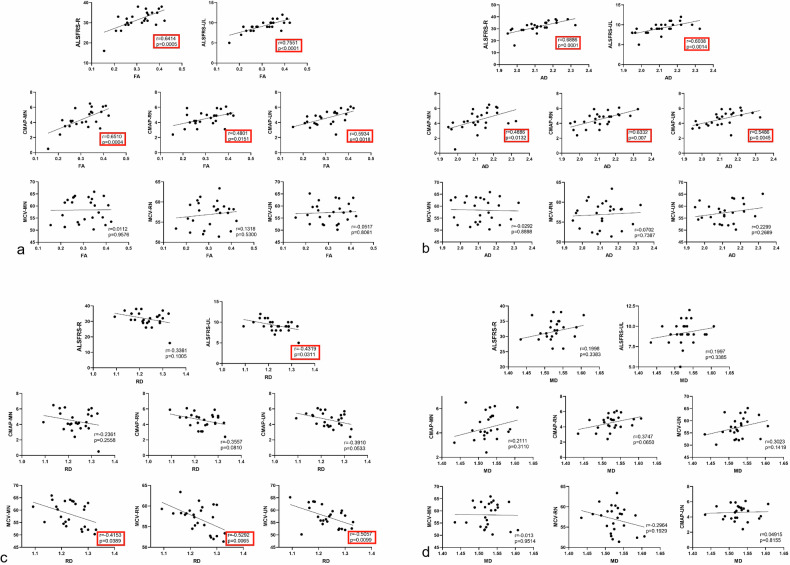
Correlations between diffusion tensor imaging parameters of brachial plexus nerve roots and ALSFRS-R score, ALSFRS-UL score, electrophysiological of median nerve (MN), ulnar nerve (UN) and radial nerve (RN). Correlation of FA (**a**), AD (**b**), RD (**c**), and MD (**d**) with ALSFRS-R score, ALSFRS-UL score, electrophysiological of MN, UN and RN, respectively. AD, Axial diffusivity; ALSFRS-R, Amyotrophic lateral sclerosis functional rating scale-revised; ALSFRS-UL, ALSFRS-upper limb; CMAP, Compound muscle action potential; FA, Fractional anisotropy; MCV, Motor nerve conduction velocity; MD, Mean diffusivity; RD, Radial diffusivity

For clinical scores, the FA and AD values showed a positive correlation with ALSFRS-R score and ALSFRS-UL score. The RD values showed a negative correlation with the ALSFRS-UL score. No significant correlation was found between RD and ALSFRS-R score and between MD and ALSFRS-R score or ALSFRS-UL score. For electrophysiological parameters, the FA values showed a positive correlation with CMAP amplitude of MN, RN and UN. The AD values showed a positive correlation with the CMAP amplitude of MN, RN, and UN. The RD values showed a negative correlation with MCV of MN, RN, and UN. No correlation was found between FA and MCV, and between AD and MCV. No correlation was found between RD and CMAP amplitude. No correlation was found between MD and CMAP amplitude and between MD and MCV. The statistical results are shown in Table 4 and Fig. 4

**Table 4:** Correlation between DTI parameters with clinical and electrophysiological measures in ALS patients

DTI parameter	ALSFRS-R score		ALSFRS-UL score		CMAP amplitude of MN		CMAP amplitude of RN		CMAP amplitude of UN		MCV of MN		MCV of RN		MCV of UN	
	*r*	*p*	*r*	*p*	*r*	*p*	*r*	*p*	*r*	*p*	*r*	*p*	*r*	*p*	*r*	*p*
FA	0.641	**<** **0.001**	0.755	**<** **0.001**	0.651	**<** **0.001**	0.480	**0.015**	0.593	**0.002**	0.011	0.958	0.132	0.530	-0.052	0.806
AD	0.689	**<** **0.001**	0.604	**0.001**	0.489	**0.013**	0.633	**0.007**	0.549	**0.005**	-0.030	0.890	0.070	0.739	0.230	0.269
RD	-0.336	0.101	-0.432	**0.031**	-0.236	0.256	-0.356	0.081	-0.391	0.053	-0.415	**0.039**	-0.529	**0.007**	-0.506	**0.010**
MD	0.120	0.338	0.120	0.339	0.211	0.311	0.375	0.065	0.302	0.142	-0.013	0.951	-0.296	0.193	0.049	0.816

Bolded values indicate statistically significant differences (*p* < 0.05). *AD* Axial diffusivity, *ALS* Amyotrophic lateral sclerosis, *ALSFRS-R* Amyotrophic lateral sclerosis functional rating scale-revised, *ALSFRS-UL* ALSFRS-upper limb, *CMAP* Compound muscle action potential, *DTI* Diffusion tensor imaging, *FA* Fractional anisotropy, *MCV* Motor nerve conduction velocity *MD* Mean diffusivity, *RD* Radial diffusivity

## Discussion

In this study, we detected characteristic changes of DTI parameters in brachial plexus nerve roots of ALS patients and further assessed their correlations with clinical and electrophysiological parameters. The agreement between observers was excellent for all DTI parameters. In ALS patients, a notable reduction in FA and AD values and a significant increase in RD value were observed in the brachial plexus nerve roots when compared to healthy controls. Furthermore, FA and AD values showed a positive correlation with ALSFRS-R, ALSFRS-UL scores, and the CMAP amplitude of the MN, RN, and UN in ALS patients. RD values showed a negative correlation with ALSFRS-UL score and MCV of MN, RN and UN. These findings suggest that DTI parameters effectively reflect the microstructural changes in the brachial plexus nerves of ALS patients, offering a non-invasive and viable quantitative approach to enhance the understanding of peripheral nerve damage in ALS.

To date, the majority of studies have concentrated on morphological and signal intensity changes observed in T2-weighted sequences [8, 9], while limited research has explored the alterations in DTI within the brachial plexus nerves of ALS patients. Currently, the application of DTI in peripheral nerves is becoming increasingly widespread, with a growing body of literature confirming its significant value in the evaluation of peripheral nerve diseases [12, 15]. Few investigations have applied peripheral nerve DTI in ALS, with most focusing on the nerves of the four extremities rather than the spinal nerve roots, particularly the brachial plexus roots. This study is the first to investigate changes in DTI parameters of the brachial plexus nerve roots in patients with ALS, and this study integrates the correlation analysis between DTI parameters and clinical scores as well as electrophysiological indicators, offering a more comprehensive perspective. Simon et al [16] reported lower FA and AD values at baseline in ALS patients in the tibial and peroneal nerve, which was in line with our study. The primary neurophysiological alterations in ALS within the peripheral nerves are characterized by progressive axonal degeneration and the loss of motor nerve fibers [17, 18]. FA reflects the physiologic anisotropy of peripheral nerves. Any nerve injury, including the damage of fiber integrity and physiologic anisotropy, can cause a decrease in FA [19].

In this study, the decrease in FA values of the brachial plexus nerves in ALS patients may be related to pathological changes such as reduction or loss of nerve fibers. AD values indicate the extent of diffusion along the longitudinal axis of fiber bundles, whereas RD values represent the degree of diffusion occurring transversely across the long axis of the fiber bundles [19]. AD is commonly regarded as an indicator of axonal integrity [19], so in our study, the reduction of AD may be related to axonal degeneration in ALS. In contrast to the findings of Simon et al regarding DTI studies of the tibial and common peroneal nerves, our research results indicate a significant increase in the RD values of the brachial plexus nerves in patients with ALS. RD is considered a reflection of the integrity of the nerve sheath structures [20]. Several studies have reported the presence of demyelination as a pathological alteration during the progression of ALS, evidenced by histological abnormalities such as myelin defects and the accumulation of pathological protein aggregates in oligodendroglial cells, as observed in both ALS patients and animal models of the disease [21]. Thus, the RD of brachial plexus nerve roots was significantly increased. We speculated that demyelination was more pronounced in proximal nerve roots than in the peripheral nerves of the four extremities in ALS. Further research is needed to elucidate the actual mechanism.

As mentioned above, in ALS, both axonal damage and demyelination are concurrently present, leading to a reduction in AD and an increase in RD. The changes in MD are determined by the combined effects of AD and RD, reflecting the overall pathological alterations in the tissue [22]. In this study, the absence of significant changes in MD may be attributed to the counterbalancing effects of decreased AD (due to axonal injury) and increased RD (due to demyelination), resulting in a net stabilization of MD values. This highlights the complexity of microstructural changes in ALS and underscores the importance of analyzing both AD and RD independently to gain a comprehensive understanding of the underlying pathology.

Further investigation into the correlation between DTI parameters and electrophysiological markers revealed that both FA and AD values were positively correlated with the CMAP amplitude of the MN, RN, and UN in ALS patients and RD values were negatively correlated with MCV of MN, RN and UN. Neurological and electrophysiological assessments are crucial for identifying peripheral nerve damage, as they provide functional insights into nerve health by analyzing MCV and CMAP amplitude. Typically, a reduction in MCV indicates demyelination, while a decreased CMAP amplitude suggests axonal damage in peripheral nerves. Since the fibers from the C5 to C8 nerve roots contribute to the formation of MN, RN, and UN, the findings of this study imply that lower FA and AD values are associated with greater axonal damage and higher RD values are associated with greater myelin damage in these nerves. So, FA, AD and RD values of the nerve roots reflect the conduction function of the MN, RN, and UN. Additionally, magnetic resonance neurography combined with DTI offers a clear advantage over electrophysiological assessments, particularly when evaluating plexuses located deeper within the body [23]. FA, AD and RD values can be suggested as appropriate surrogate measures to reflect the functional changes of nerves for ALS patients, especially for patients inconvenient for an electrophysiological examination.

The results of this study indicate that FA and AD values are positively correlated with clinical symptom scores, while RD values are negatively correlated with upper limb clinical symptom scores. In other words, lower FA and AD values are associated with more severe clinical symptoms, whereas higher RD values correspond to more severe upper limb clinical symptoms. Therefore, FA, AD, and RD may have certain clinical value in evaluating the severity of ALS and monitoring disease progression, and may serve as useful objective imaging biomarkers for evaluating treatment efficacy. Future studies with larger sample sizes are needed to further validate the clinical utility of DTI parameters in the diagnosis and monitoring of ALS.

This study has some limitations. First, the study’s sample size was limited, which may be attributed to the relatively low incidence of ALS with upper extremity onset and the exclusion of patients with severe symptoms who could not tolerate MRI examinations. This small sample size reduces statistical power and increases the risk of both Type I and Type II errors, potentially affecting the reliability of both our positive and negative findings. The limited power may particularly influence mean estimates of diffusivity metrics *e.g*., MD values), as evidenced by their wide dispersion in our cohort. While we observed strong correlations between some DTI parameters and electrophysiological measures, these results require cautious interpretation and validation in larger samples. Future research should aim to expand the sample size through multi-center collaborations to improve the generalizability of findings. Secondly, this study did not conduct longitudinal tracking studies on ALS patients. Future studies should conduct longitudinal studies to further explore the correlation between DTI parameter values and the progression of ALS. Thirdly, the current study lacked histopathological confirmation of peripheral nerves in ALS patients. Future studies incorporating nerve biopsies or postmortem examinations will be essential to validate our imaging findings and elucidate the correlation between imaging features and underlying pathological changes. Fourthly, in this study, DTI post-processing was performed using the Philips ISP workstation, a dedicated and standardized research platform provided by Philips. While this approach ensures reproducibility within the Philips ecosystem, the use of open-source alternatives (*e.g*., FSL, MRtrix) could facilitate broader cross-platform validation. Future studies may consider incorporating such tools to further assess the generalizability of the findings.

In conclusion, our preliminary findings suggest that FA, AD, and RD values from DTI may have the potential to identify microstructural changes in the brachial plexus nerve roots of ALS patients. The observed correlations with electrophysiological markers, while requiring further validation in larger cohorts, tentatively indicate that these DTI parameters could serve as candidate indicators of nerve conduction function in the upper extremities. However, given the limited sample size of this study, these results should be interpreted as exploratory rather than definitive, and future studies with larger sample sizes are needed to confirm these relationships.

## Data Availability

The data that support the findings of this study are available from the corresponding author, Guangbin Wang, upon reasonable request.

## Abbreviations

AD: Axial diffusivity
ALS: Amyotrophic lateral sclerosis
ALSFRS: ALS Functional Rating Scale
AUROC: Area under the receiver operating characteristic curve
DTI: Diffusion tensor imaging
FA: Fractional anisotropy
MCV: Motor nerve conduction velocity
MD: Mean diffusivity
MN: Median nerve
RD: Radial diffusivity
RN: Radial nerve
ROC: Receiver operating characteristic
ROI: Region of interest
SHINKEI: SHeath signal increased with INKed rest-tissue RARE Imaging
UN: Ulnar nerve
